# A Chair-Based Music–Kinetic Combined Exercise Program as an Alternative Approach for Increasing Health, Functional Capacity, and Physical Fitness Indices in Middle-Aged Pre-Menopausal Women

**DOI:** 10.3390/jfmk8020081

**Published:** 2023-06-15

**Authors:** Konstantina Karatrantou, Theodoros Papavasiliou, Christos Batatolis, Theodora Vasilopoulou, Panagiotis Ioakimidis, Vassilis Gerodimos

**Affiliations:** Department of Physical Education and Sports Science, University of Thessaly, 42100 Trikala, Greecevasilopoulouphys@gmail.com (T.V.); ioakeimidis@uth.gr (P.I.);

**Keywords:** aerobic dance, flexibility, balance, strength, integrated concurrent training, aging

## Abstract

Lately, chairs have been widely used as a cheap, easily accessible, safe, and effective training means in different settings (e.g., in gyms, the house, workplaces, and in rehabilitation). This study investigated the effectiveness of a 10-week chair-based music–kinetic integrated combined exercise program on health, functional capacity, and physical fitness indicators of middle-aged pre-menopausal women. A total of 40 healthy women (40–53 years) were assigned to two groups: exercise (EG) and control (CG). The EG followed a 10-week (3 times/weekly; 30 training sessions) chair-based exercise program including aerobic dance, flexibility, coordination, and strength exercises with body weight or auxiliary means. Selected indicators of health, functional capacity, and physical fitness were evaluated before and after the 10 weeks. Following the program, the EG significantly reduced their body fat (−2.5%), blood pressure (by −4.5 to −5.5%), the time during the timed up-and-go (TUG) test (by −10.27%), heart rate (by −6.35 to −13.78%), and the rate of perceived exertion (by −24.45 to −25.88%), while increasing respiratory function (3.5–4%), flexibility (12.17%), balance (50.38–51.07%), maximal handgrip strength (10–12.17%), and endurance strength (43.87–55.91%). The chair-based combined music–kinetic exercise program was effective and could be safely used in different settings to improve health, functional capacity, and physical fitness in middle-aged women.

## 1. Introduction

Middle age (40–64 years old) is one of the most important periods of women’s life and marks the transition from young adulthood to older age [1]. This period of life is associated with various physical, mental, cognitive, and social changes, which affect women’s health-related quality of life (HRQOL) [1,2]. The World Health Organization (WHO) defines HRQOL as “perfect physical, mental, and social health and well-being”, which is affected by different daily lifestyle behaviors [3]. The reduced physical activity and the adoption of other unhealthy behaviors (i.e., smoking, alcohol consumption, and unhealthy eating habits) are considered important risk factors related to (a) the development of chronic diseases (obesity, hypertension, cardiovascular diseases, diabetes, etc.), (b) a reduction in independent living, (c) the deterioration of HRQOL, and (d) the decrease of life expectancy in middle-aged and older individuals [4,5,6]. Despite the current recommendations of the WHO and the American College of Sports Medicine (ACSM) for regular physical activity and exercise to improve or maintain physical and mental health, many middle-aged and older individuals have not adopted a physically active lifestyle [4,5]. The low availability and accessibility of safe exercise programs and training means according to the individual’s possibilities, high costs and time constraints are considered the main contributing factors to the reduced participation of the population in exercise activities [4,6]. Taking all the above into consideration, several organizations all over the world suggest the design and implementation of appropriate exercise programs for untrained middle-aged and older individuals [4,5,6], using safe, cheap, easily applicable, and accessible training means.

For this reason, during the last few decades, chairs have been used as an alternative training means for implementing various exercise programs (flexibility, balance, strength, and aerobic capacity) in different populations (i.e., healthy older individuals, young and middle-aged employees, individuals with chronic diseases or mobility difficulties, pregnant women, and untrained individuals) [7,8,9,10,11,12,13,14,15,16,17,18,19,20,21,22] and have several advantages. First of all, the use of a chair minimizes the risk of falls and injury during exercise by offering better stability, support of the human body, and safety to the trainees [7,10,15]. Other important advantages of chairs are their low cost and easy accessibility. Moreover, exercise programs using chairs as a basic training means can be easily adapted and implemented in any indoor or outdoor place (without requiring particularly large space), such as in the gym, at home, in the workplace, and in a classroom, courtyard, park, rehabilitation center, or hospital room. In the scientific literature, several studies have examined the effect of chair-based exercise programs in different populations reporting promising results in various cardiovascular and neuromuscular health-related parameters (i.e., gait, balance strength, flexibility, blood pressure, heart rate, and musculoskeletal pain) [7,8,9,10,11,12,13,14,15,16,17,18,19,20,21,22]. It should be mentioned that the vast majority of the aforementioned studies (a) were performed in elderly frail people or individuals with chronic diseases [7,8,9,10,11,12,15,16,17,18,19,20,21,22], while there is limited information on healthy middle-aged individuals and (b) were focused on improving single or specific indices of health and physical fitness. To the best of our knowledge, no previous study has examined the effectiveness of a “holistic” chair-based exercise approach focusing on all the important functional and physical fitness parameters (aerobic capacity, strength, flexibility, and coordination abilities). However, according to the ACSM and the WHO, middle-aged individuals should engage in specifically designed combined exercise programs at least 3 times per week, which should entail cardiorespiratory and neuromuscular exercises (balance, strength, and flexibility) aiming to improve or preserve physical fitness and health [4,5].

Furthermore, to the best of our knowledge, no previous study has investigated the effectiveness of a music–kinetic chair-based exercise program in healthy middle-aged individuals, while several studies have used music–kinetic exercise programs (different dance training programs) without a chair, reporting beneficial effects in physical and mental health in middle-aged individuals [23,24,25,26,27,28]. During the last few decades, several studies have addressed the impact of music either as an ergogenic aid or as a means by which to adjust and control the intensity of exercise, causing the appropriate adaptations to the human body [29,30]. Previous studies in the scientific literature have reported a significant interaction between exercise intensity (heart rate and rate of perceived exertion responses throughout exercise workout) and music tempo in different exercise modalities (e.g., cycling and treadmill walking) [31,32]. Additionally, the use of music throughout exercise programs has been shown to (a) reduce the perception of fatigue and exertion through dissociation and distraction during exercise, (b) increase arousal and neural activity, and as a result, improve exercise performance, and (c) increase motivation and effort, mood, exercise enjoyment, and feelings of power [33,34,35,36,37]. For this reason, it is of interest to design, implement and evaluate the efficacy of a music–kinetic chair-based combined exercise program that can be used in different exercise and rehabilitation settings to improve health, functional capacity, and overall fitness in middle-aged untrained individuals.

Thus, the present study aimed to examine the effectiveness of a 10-week chair based music–kinetic integrated (where the exercise goals were repeatedly altered during the main part of the training session) combined exercise program (aerobic dance, flexibility, coordination, and strength exercises with body weight or auxiliary means) on health (body fat, blood pressure, and respiratory function), functional capacity (lower back and hamstrings flexibility, static and dynamic balance), and physical fitness (strength and aerobic capacity) indicators of healthy middle-aged pre-menopausal women.

## 2. Materials and Methods

### 2.1. Participants

Firstly, 50 premenopausal middle-aged women, following announcements in mass media (e-magazines and e-newspapers) and on social media (Facebook and Twitter), were recruited to participate in this study and were assessed for eligibility. Six of them were excluded because they did not meet some of the inclusion criteria (they had chronic health problems, such as diabetes, cardiovascular disease, and hypertension) and four of them were excluded because they were unable to complete the initial functional capacity and physical fitness measurements due to musculoskeletal discomfort in the knee joint and the lumbar spine. Thus, the final sample of the present study was 40 pre-menopausal middle-aged untrained women (40–53 years old), who were divided into two equal groups: the exercise group (EG; n = 20) and the control group (CG; n = 20) (Table 1). Before the study, the subject’s medical history and activity status were assessed using a specific questionnaire. All the final participants of the present study (a) were healthy without injury or disease and were free of any illness, (b) did not report the use of any medication and (c) did not participate in any organized physical activity for at least six months before the study. In addition, before the study, the participants were informed about the experimental procedures and the possible risks during the study and signed an informed consent form. The conduction of the study took place according to the Declaration of Helsinki and ethical approval was granted by the Ethics Committee of the University of Thessaly.

### 2.2. Study Design

Primarily, a pilot study was performed to determine the final testing and training procedures. One to two weeks before the start of the study, the participants performed two–three familiarization sessions to become accustomed to the instrumentation and the experimental (testing and training) procedures. Subsequently, the baseline measurements were performed in the total sample. Following the pre-training measurements, the participants were randomly assigned to two equal groups: the EG and the CG. A computer-generated list of random numbers was used for the allocation of the sample in one of the two groups, exercise or control. It should also be mentioned that the main investigator and the outcome assessors were blinded to the allocated intervention during the entire period of data collection. During the study, the EG participated in a 10-week (3 times per week) music–kinetic chair-based integrated combined training program; while the CG did not perform any systematically organized training program during the 10 weeks. It would be important to mention that no adverse effects or injuries were reported during the 10-week exercise program. The training program was supervised by an exercise instructor, and special exercise attendance books were filled in to confirm the participants’ exercise adherence (all of the participants of the study completed 30 training sessions in total). Exercise adherence was also reinforced using different motivation strategies (i.e., think positive, make a commitment, and set short-term goals) [38]. Two days after the end of the training session, the pre-training measurements were repeated in the same order and at the same time of day. The EG was instructed not to engage in any other activities during the research. In addition, the participants were instructed to continue their regular diet during the study, avoid the consumption of caffeine, alcohol, and tobacco at least 24 h before the test, avoid any intense activity before the test, and rest sufficiently the night before the test.

### 2.3. Training Program

The EG followed a 10-week (3 days/week; 30 training sessions in total) music–kinetic integrated combined chair-based exercise program (flexibility, balance, strength, and aerobic capacity). Each training session lasted about 50–60 min and included 15-min warm-up and flexibility training, a 30–40-min main part (integrated combined balance, strength, and aerobic training using chair-based seated and chair-assisted standing exercises), and 5-min recovery. During the main part of the training program, the balance, strength, and aerobic training were altered in a predetermined order (2–2.5-min aerobic dance/1–1.5-min strength or balance exercises). Throughout each exercise session of the 10-week exercise program, real-time monitoring of the participants’ heart rate was performed using the Polar Team Solution system (Science Technologies, Kempele, Finland), and the rating of perceived exertion (RPE) was assessed using the 20-point Borg scale after each exercise session to ensure the safety of the participants. The basic training mean of the program was the chair. It should be mentioned that special emphasis was placed on the selection of the appropriate chair used for the program. In more detail, the criteria for choosing the appropriate chair were as follows: (a) a stable chair without wheels, (b) a chair without armrests so that the movements are not hindered, (c) a chair with a back for better lumbar spine support (not a softback), and (d) the chair height adjusted depending on the height of the participants, so that the feet could be flat on the floor with a knee angle approximately at 90°.

The main part of the training program included:

(a) Aerobic training: The aerobic training program included low-impact aerobic dance movements from a sitting position in the chair or from an upright position around the chair (i.e., knee lift, heel up, kick, lateral lunges, squats, and V step) in conjunction with continuous arm movements at the shoulder level as well as above the head. We selected a low-impact aerobic dance because it is safe and is widely used in fitness and rehabilitation centers for improving indices of health and overall fitness in middle-aged and older individuals. During the aerobic dance choreography, the women hold a medium resistance anti-stress ball or a hand gripper in each hand and squeezed it simultaneously according to the specific aerobic dance movements. The intensity (65–80% of the age-predicted HR_max_; music rhythm 110–120 beats/min) and the duration (16–25 min) of aerobic training progressively increased during the training program, according to the recommendation of the ACSM [5].
(b) Strength training: The strength training program included chair-based seated and chair-assisted standing exercises with body weight as well as exercises with auxiliary means (dumbbells, Pilates mini balls, rings, anti-stress balls, and hand grippers) for all major muscle groups. In more detail, the program consisted of exercises for the lower limbs (i.e., sit-to-stand exercise, adductor ball squeeze from sitting position, standing lateral hip raises with chair support, and calf raises with chair support), the upper limbs (i.e., tricep extensions, lateral raises, and bicep curls from the sitting position), as well as for the abdominal and dorsal trunk muscles (i.e., modified chair sit-ups, sitting twists, the seated cat–cow exercise, and seated knee lifts). The training load of strength exercises, throughout the 10-week exercise program, was modified by progressively increasing the number of sets (1–3), repetitions (8–15 RM), and the resistance of the dumbbells (1–3 kg), according to the recommendation of the ACSM [5]. All strength exercises were performed following the music rhythm (100–110 beats/min).
(c) Balance training: The balance training program included static (two-leg and one-leg stance exercises) and dynamic balance exercises with dynamic motion in lower and/or upper limbs (i.e., calf raises, standing hip extensions, and standing hip abductions) as well as dynamic balance exercises with different ways of locomotion (i.e., heel-to-toe forward walking, backward walking, lateral walking, heel walking, and toe walking). The static balance exercises were initially performed, with the support of one or two hands on the chair and without auxiliary means, and then performed without the support of the hands on the chair using small auxiliary means (Pilates mini balls and rings, anti-stress balls, and hand grippers). The training load of balance exercises, throughout the 10-week exercise program, was modified by progressively increasing the number of sets (1–3), the repetitions/duration (8–15 reps or 10–30 s), and the distance in dynamic balance exercises with locomotion (3–5 m), according to the recommendation of the ACSM [5]. All balance exercises were performed following the music rhythm (100–110 beats/min).

The training load characteristics during the 10-week intervention are analytically presented in Table 2.

Additionally, an indicative training session (the ninth training session) of the 10-week intervention program is analytically presented in Table 3.

### 2.4. Testing Procedures

Health, functional capacity, and physical fitness indices were measured in both the EG and the CG before and after the 10 weeks. Before the functional capacity and physical fitness testing, a 10-min warm-up was performed by the participants including 5-min stationary cycling and 5-min static-dynamic stretching exercises for all major muscle groups.

#### 2.4.1. Health Indices

(a) The percentage of body fat (%BF) was assessed using the bioelectrical impedance method (Maltron 900) and then the fat-free mass (FFM) was calculated; (b) blood pressure and (c) respiratory function (FVC; FEV_1_) were also measured using an electronic blood pressure monitor (A&D-UA-851) and a portable spirometer (Micro, Medical, Micro), respectively [39,40].

#### 2.4.2. Functional Capacity Indices

The lower back and hamstrings’ flexibility was assessed with the sit and reach test using a special box (sit and reach flex tester, Novel Products Inc., Rockton, IL, USA) following the instructions of the ACSM [34]. The participants were instructed to lean forward slowly as far as possible (while exhaling), without bending their knees, and to hold at the final position for at least 2 s [39]. The participants performed three trials with a rest period of 15 s, and the best score (in cm) was considered for analysis, while the static and dynamic balance were also evaluated using the 1-min single limb stance and the timed up and go test, as previously described by Douris et al. [41] and Rikli and Jones [42]. During the static balance test, the participants were asked to stand on a firm surface and look straight ahead. The stopwatch began after the participants raised one leg and stopped when the participants became unstable and placed the flexed leg on the ground or at the completion of 1-min [41]. Both legs were tested in a random order, and the mean score (in s) of three trials of each leg was considered for analysis. While during the TUG test, the participants were instructed to stand up from a chair, walk as quickly as possible for 3 m, turn around, walk back, and sit down in the chair [42]. The participants performed three trials (with 30 s rest between the trials), and the best score (in s) was considered for analysis.

#### 2.4.3. Physical Fitness Indices

The muscular strength and endurance of the upper body were assessed using three reliable tests: (a) the maximal handgrip strength test, (b) the sit-ups test, and (c) the modified knee push-ups test, which are widely used in the scientific literature using different testing protocols and equipment for the evaluation of various populations such as young and middle-aged adults, children, and adolescents [39,43,44,45]. In more detail, the maximal handgrip strength test was conducted with a hydraulic dynamometer (Jamar 5030J1, Jamar Technologies, Horsham, PA, USA) and performed from the sitting position on a height-adjustable chair with the participants’ feet supported, their shoulders adducted and neutrally rotated, their elbow flexed at 90°, and their forearm in the neutral position [43]. The participants performed three maximal isometric contractions (each lasting 5 s) with each hand, with a 1-min rest between trials, and the best score with each hand was considered for analysis. Furthermore, during the sit-up test, the participants were instructed to lie in the supine position with their knees bent at an angle of 100–110° and with both arms folded across their chest. The maximum number of sit-ups was considered for analysis [39]. Additionally, during the push-ups test, the participants were positioned with their knees bent at an angle of 100–110° and their arms extended at a shoulder-width apart. The participants lowered their bodies, bending their elbows at an angle of 90°, and then returned back to the starting position. The maximum number of push-ups was considered for analysis [39].

Finally, cardiorespiratory fitness was assessed using the submaximal treadmill walking test of Ebelling et al., consisting of three 4-min stages [46]. The participants started the 4-min walking test protocol at an initial treadmill velocity that corresponded to 60% of their age-predicted HR_max_ and 0% grade (stage 1). Then, the walking speed remained stable for each participant, and the treadmill incline was increased to 5% (stage 2) and 10% (stage 3). The heart rate of the participants was measured before the walking test protocol, after each stage, and at the first minute following the termination of the walking test using chest belt telemetry (Polar Electro, Kempele, Finland). Additionally, the rating of perceived exertion (RPE) was assessed at the end of each stage using the 20-point Borg scale. Maximal oxygen uptake (VO_2max_; mL/kg/min) was also estimated using the following equation proposed by Ebbeling et al. [46]: VO_2max_ = 15.1 + 21.8 (speed in mph) −0.327 (heart rate in bpm at 5% grade) −0.263 (speed × age in years) + 0.00504 (heart rate in bpm at 5% grade × age in years) + 5.98 (gender: female = 0, male = 1).

### 2.5. Statistical Analysis

All statistical analyses were performed using IBM SPSS Statistics v.26 software (IBM Corporation, Armonk, New York, USA), and the results are presented as the means ± standard deviations. A statistical power analysis (software package GPower 3.0) before the initiation of the study indicated that a total number of 36 participants (18 participants in each group) would yield adequate power (>0.85) and a level of significance (<0.05). The total sample of the present study was 40 middle-aged pre-menopausal women (20 participants in each group). The normality of data was examined using the Shapiro–Wilk test (all variables followed the normal distribution). Two-way analysis of variance (ANOVA), 2 groups (EG and CG) × 2 time points (pre and post training), with repeated measures on the “time point” factor and multiple comparisons with the Sidak method, was applied to locate significantly different means within and between the groups. Cohen’s effect sizes were calculated using the equation: d = difference between means/pooled SD. One-way ANOVAs were used between the groups to compare the relative changes from pre to post training in all of the tested parameters. The significance level was set at *p* < 0.05.

## 3. Results

### 3.1. Health Indices

Two-way ANOVAs indicated significant interaction effects on the percentage of body fat and blood pressure as well as on respiratory function (FVC; FEV_1_) (*p* < 0.05). Specifically, the EG body fat and blood pressure values were significantly lower in the post-training measurements versus the pre-training measurements, while the FVC and FEV_1_ values were significantly greater in the post-training measurements versus the pre-training measurements (*p* < 0.05; with small–medium effect sizes d = 0.24–0.43), and the FFM values remained stable (*p* > 0.05). In the CG, all the above variables did not change after the 10 weeks (*p* > 0.05) (Table 4). Comparisons between groups revealed that all post-training values for body fat and blood pressure were significantly lower in the EG versus the CG, while the FVC and FEV_1_ values were significantly higher in the EG versus the CG (*p* < 0.05). The FFM post-training values did not differ between the EG and the CG (*p* > 0.05). The percent changes from pre to post training for body fat, blood pressure, and respiratory function were significantly greater in the EG vs. the CG (*p* < 0.01). Regarding the pre-training measurements, no significant differences were observed in all health parameters between the two groups (*p* > 0.05).

### 3.2. Functional Capacity Indices

The two-way analyses of variance showed significant interaction effects on all functional capacity indices (flexibility and static and dynamic balance) (*p* < 0.001). More specifically, in the EG, the flexibility and static balance values were significantly higher in the post-training measurements versus the pre-training measurements, while the TUG values were significantly lower in the post-training measurements versus the pre-training measurements (*p* < 0.001; d = 0.70–1.15). In the CG, all of the above variables did not change after the 10 weeks (*p* > 0.05) (Table 5). Comparisons between the groups revealed that all of the post-training flexibility and static balance values were significantly greater in the EG versus the CG, while the TUG values were significantly lower in the EG versus the CG (*p* < 0.001). Regarding the pre-training measurements, no significant differences were observed in all functional capacity parameters between the two groups (*p* > 0.05).

### 3.3. Physical Fitness Indices

#### 3.3.1. Strength

The analyses of variance indicated significant two-way interaction effects on all of the strength tests (*p* < 0.001). More specifically, in the EG, the handgrip, push-ups, and sit-ups test values were significantly higher in the post-training measurements versus the pre-training measurements (*p* < 0.001; d = 0.70–1.57). In the CG, the above variables did not change after the 10 weeks (*p* > 0.05) (Table 6). Comparisons between the groups revealed that all post-training strength values were significantly greater in the EG versus the CG (*p* < 0.001). The percent changes from pre to post training for the handgrip, push-ups, and sit-ups test were significantly greater in the EG vs. the CG (*p* < 0.001). Regarding the pre-training measurements, no significant differences were observed in all the upper body strength values between the two groups (*p* > 0.05).

#### 3.3.2. Aerobic Capacity

The analyses of variance demonstrated significant two-way interaction effects on the heart rate and RPE values (*p* < 0.001; Table 7). Specifically, the EG heart rate and RPE values were significantly lower in the post-training measurements versus the pre-training measurements (*p* < 0.001; d = 0.7–1.15), while the VO_2max_ values were significantly higher. In the CG, the above variables did not change after the 10 weeks (*p* > 0.05). Comparisons between the groups revealed that all heart rates and RPE post-training values were significantly lower in the EG versus the CG (*p* < 0.001), while the VO_2max_ values were significantly higher. The percent changes from pre to post training for heart rate, RPE, and VO_2max_ were significantly greater in the EG vs. the CG (*p* < 0.001). Concerning the pre-training measurements, non-significant differences were observed between the two groups (*p* > 0.05).

## 4. Discussion

Biological aging in conjunction with physical inactivity and reduced participation in organized exercise programs may lead to a decline in cardiovascular and neuromuscular function, contributing to physical frailty and deterioration in health-related quality of life [4,5,47,48]. For this reason, all health and exercise organizations such as the ACSM, the WHO, and the CDC recommend the systematic participation of middle-aged and older individuals in combined exercise programs consisting of cardiovascular and neuromuscular activities as the most effective “non-pharmacological” intervention for counteracting the harmful effects of a sedentary lifestyle and aging [4,5,6]. In this context, during the last few decades, different sports and health professionals all over the world have focused on the design, implementation, and evaluation of different serial and integrated combined exercise programs using various activities and training means [13,14,23,24,26,28,49,50,51,52,53,54,55]. Although chair-based exercise programs have gained popularity as an alternative mode of exercise for improving health, functional capacity, and physical fitness, especially in older and frail individuals [7,8,9,10,11,12,15,16,17,18,19,20,21,22], only a few studies have examined the effects of chair-based exercise programs in healthy middle-aged individuals [13,14]. This study was designed and implemented with success and safety (without adverse side effects or injuries) in middle-aged pre-menopausal women with a 10-week supervised music–kinetic chair-based integrated combined exercise program using chair-based seated and chair-assisted standing flexibility, balance, and strength exercises as well as low-impact aerobic dance movements. The exercise program that was implemented was effective in inducing significant adaptations to all health, neuromuscular, and cardiovascular parameters.

Neuromuscular function (flexibility, balance, and strength) plays an important role in the safe and effective participation of middle-aged and older individuals in daily activities [5]. The progressive loss of flexibility, balance, and strength is an inevitable occurrence of aging [5], contributing to physical frailty and a deterioration in health-related quality of life. This study showed that a 10-week supervised music–kinetic integrated combined exercise program using chair-based seated and chair-assisted standing flexibility, balance, and strength exercises with body weight or small auxiliary means (dumbbells, Pilates mini balls, and rings, anti-stress balls, and hand grippers) resulted in a 12% gain in flexibility of the hamstring and/or lower back muscles, a 50–51% gain in static balance, a 10% gain in dynamic balance, a 10–13% gain in maximal handgrip strength, and a 44–56% gain in upper body endurance strength. The findings of the present study are in line with previous investigations reporting similar gains in neuromuscular parameters (flexibility or static and dynamic balance or lower and upper body strength) after different chair-based exercise programs in older and frail individuals with chronic diseases or injuries [7,8,9,10,11,12,15,16,17,18,19,20,21,22] as well as in employees of different working environments (i.e., offices and hospitals) [13,14]. The results of previous studies that implemented different combined exercise programs without chairs demonstrate conflicting results. Some of them following the results of the present study reported significant improvements in neuromuscular indices of middle-aged men and women, while others failed to observe significant neuromuscular training adaptations [23,24,27,49,50,56]. According to several previous investigators, the confusing results among studies concerning neuromuscular adaptations may be attributed to differences in subjects’ characteristics, loading parameters, and exercise modalities, but mainly to the order of exercises, which may reinforce the so-called “interference effect”, diminishing the efficacy of combined strength and aerobic training compared to separately training only strength or endurance [57,58].

Strength and endurance training activate different mechanisms, therefore causing opposite adaptations to the human body. In more detail, strength training causes skeletal muscle hypertrophy and neuromuscular responses by activating the mammalian/mechanistic target of the rapamycin (mTOR) signaling pathway [59,60], whereas aerobic training causes skeletal muscle oxidative and metabolic capacity [61] by activating adenosine monophosphate (AMP)-activated protein kinase (AMPK). It should be mentioned that AMPK interferes with mTOR signaling via tuberous sclerosis complex 2 (TSC2), repressing protein synthesis [62]. Taking all the above into consideration, the combination of those two different modes of training (strength vs. aerobic) in the same training period/session may lead to the so-called “interference effect”. Some previous studies have reported that residual fatigue produced by prior aerobic exercise workouts decreases the neural input to the exercised muscle leading to decrements in force output and the rate of force development, as well as attenuation of neuromuscular responses [52]. Conversely, some other studies have demonstrated that the mode of combined training (serial or integrated) and the order of exercises during combined training does not affect neuromuscular responses [53]. In our study, we chose an integrated combined training mode, which according to previous studies may reduce or eliminate the “interference effect” between neuromuscular and aerobic exercise workouts due to less muscle soreness and faster muscle recovery after exercise [63,64,65].

Cardiorespiratory fitness (CRF) is also an important indication of a person’s overall physical health, and it refers to the capacity of the circulatory and respiratory systems to supply oxygen to skeletal muscle mitochondria for the energy production needed during physical activity [53,54]. Low levels of CRF in conjunction with high levels of body fat are strong predictors of cardiovascular disease (CVD) and all-cause mortality in middle-aged and older individuals [66,67]. The present study reported that a music–kinetic chair-based integrated combined exercise program decreased body fat by 2.5% (with a small effect size), blood pressure by 4.5–5.5% (with a small effect size), heart rate by 6.5–14%, and the rate of perceived exertion by 25–26%, while it increased respiratory function by 3.5–4% in middle-aged pre-menopausal women. To the best of our knowledge, very few studies [13,14,68] have examined the efficacy of chair-based exercise programs on body composition and cardiorespiratory function. The results of these studies are in line with those of the present study, reporting significant benefits in body composition and cardiorespiratory fitness of employees or frail older individuals [13,14,68]. Furthermore, the results of the present study revealed similar cardiorespiratory adaptations in comparison with other studies that implemented non-chair combined exercise programs in middle-aged men and women using various activities [23,25,26,27,50].

The findings of this study are limited to the use of a 10-week music–kinetic chair-based integrated combined exercise program consisting of flexibility, balance, strength, and low-impact aerobic dance exercises using body weight or small auxiliary means (dumbbells, Pilates mini balls, and rings, anti-stress balls, and hand grippers). Future studies could investigate possible training adaptations using other exercise modalities and training interventions of greater program duration (above 10 weeks). Furthermore, our findings are limited to healthy middle-aged pre-menopausal women. Future studies could examine the efficacy and safety of music–kinetic chair-based integrated combined exercise programs in obese individuals, individuals of other age groups (i.e., children or young adults), individuals with different training statuses (trained individuals) as well as in women with different menopause stages (menopause or post-menopause). Finally, in upcoming studies, it would be interesting to examine the efficacy of the chair-based music–kinetic combined exercise program and other health-related factors such as biochemical indicators and evaluation of stress and psychological well-being.

## 5. Conclusions

In conclusion, a 10-week music–kinetic integrated combined exercise program, using chair-based seated and chair-assisted standing flexibility, balance, and strength exercises as well as low-impact aerobic dance movements, is an effective intervention that induces significant cardiovascular and neuromuscular adaptations in middle-aged pre-menopausal women, without causing adverse side effects or injuries. This study provides perspectives for an alternative and efficient exercise approach that can be used with safety in fitness and rehabilitation centers to ameliorate the age-associated loss of functional capacity and overall fitness in middle-aged women.

## Figures and Tables

**Table 1:** Age and somatometric characteristics of the middle-aged women per group (mean ± sd).

Variables	EG (n = 20)	CG (n = 20)
Age (years old)	46.80 ± 4.70	46.35 ± 3.85
Height (m)	1.64 ± 0.08	1.65 ± 0.07
Body mass (kg)	66.34 ± 7.79	67.26 ± 6.82
Body mass index (kg/m^2^)	24.66 ± 3.10	24.73 ± 3.55

EG: exercise group; CG: control group.

**Table 2:** Progression of training load during the 10 weeks.

	Weeks			
	1–2	3–4	5–7	8–10
Total duration of the training session (min)	50	50–55	57	60
Total duration of the main part (min)	30	30–35	37	40
Aerobic dance/strength balance ratio during the main part	2 min/ 1 min	2–2.20 min/ 1 min	2.5 min/ 1.20 min	2.5 min/ 1.5 min
**Aerobic training**				
Music rhythm (beats/min)	110	110–115	115	115–120
Intensity (% HRmax)	65–70%	70–75%	70–75%	75–80%
Duration (set x time)	8 × 2 min = 16 min total	10 × 2 − 2.20 min = 20–22 min total	10 × 2.5 min = 25 min total	10 × 2.5 min = 25 min total
Training contents	Seated aerobic dance	Seated aerobic dance (14 min)—standing aerobic dance around the chair (6–8 min)	Seated aerobic dance (15 min)—standing aerobic dance around the chair (10 min)	Seated aerobic dance (12.5 min)—standing aerobic dance around the chair (12.5 min)
Equipment	Anti-stress balls	Anti-stress balls	Hand grippers	Hand grippers
**Strength training**				
Music rhythm (beats/min)	100–105	105–108	108	108–110
Sets	1–2	2	2	2–3
Reps/set	8–10	10–12	12	15
Equipment	Mini balls, dumbbells 1–2 kg, and anti-stress balls	Mini balls, dumbbells 1–2 kg, and anti-stress balls	Pilates ring, dumbbells 2–3 kg, and hand grippers	Pilates ring, dumbbells 2–3 kg, and hand grippers
**Balance training**				
Music rhythm (beats/min)	100–105	105–108	108	108–110
Sets	1–2	2	2	2–3
Reps or duration (s) or distance (m)/set	8–10 reps/10–15 s/3 m	10–12 reps/15–20 s/3–4 m	12–15 reps/20–25 s/4–5 m	15–20 reps/25–30 s/5 m
Equipment	-	-	Mini balls and anti-stress balls	Pilates ring and hand grippers

**Table 3:** An indicative training session of the 10-week intervention program.

Total duration of the ninth training session: 50 min	
**Warm-up-Flexibility** (15 min)	Low-impact chair-based seated aerobic dance lower and upper limb movements (5 min). Dynamic and static stretching exercises (chair-based seated and chair-assisted standing) for the whole body (12 exercises; 2 sets × 15 s for static/15 reps for dynamic exercise).
**Main part** (30 min)	
	**Block 1**. Seated aerobic dance (2 min)/2 balance strength exercises: (a) one-leg stance with the leg bent forward at 90° (1 set × 15 s/leg) and (b) calf raises with simultaneous tricep extensions with a mini ball (1 set × 10 reps). **Block 2**. Seated aerobic dance (2 min)/2 balance strength exercises: (a) one-leg stance with the leg bent forward at 90° (1 set × 15 s/leg) and (b) calf raises with simultaneous tricep extensions with a mini ball (1 set × 10 reps). **Block 3**. Standing aerobic dance (2 min)/2 balance exercises: (a) heel-to-toe forward walking (1 set × 3 m) and (b) backward walking (1 set × 3m). **Block 4**. Seated aerobic dance (2 min)/2 balance exercises: (a) heel-to-toe forward walking (1 set × 3 m), and (b) backward walking (1 set × 3m). **Block 5**. Seated aerobic dance (2 min)/2 strength exercises: (a) lateral raises with dumbbells from a sitting position (1 set × 10 reps) and (b) bicep curls with dumbbells from a sitting position (1 set × 10 reps). **Block 6**. Standing aerobic dance (2 min)/2 strength exercises: (a) lateral raises with dumbbells from a sitting position (1 set × 10 reps) and (b) bicep curls with dumbbells from a sitting position (1 set × 10 reps). **Block 7**. Seated aerobic dance (2 min)/2 strength exercises: (a) sit-to-stand exercise (1 set × 10 reps) and (b) adductor mini ball squeeze from the sitting position (1 set × 10 reps). **Block 8**. Seated aerobic dance (2 min)/2 strength exercises: (a) sit-to-stand exercise (1 set × 10 reps) and (b) adductor mini ball squeeze from the sitting position (1 set × 10 reps). **Block 9**. Standing aerobic dance (2 min)/2 strength exercises: (a) sitting twists (1 set × 10 reps) and (b) seated cat–cow exercise (1 set × 10 reps). **Block 10**. Seated aerobic dance (2 min)/2 strength exercises: (a) sitting twists (1 set × 10 reps) and (b) seated cat–cow exercise (1 set × 10 reps).
**Cool down** (5 min)	Low-impact chair-based seated aerobic dance lower and upper limb movements (2 min) and chair-based seated static stretching exercises for the whole body in conjunction with breathing exercises (3 min).

**Table 4:** Health indices in the exercise and control group pre and post training (mean ± SD).

Variables	Group	Pre Training	Post Training	Mean % Change
Body fat (%)	EG CG	29.50 ± 6.90 29.09 ± 9.85	27.00 ± 7.50 ^*,*#*^ 29.48 ± 11.84	−2.5 ^†^ +1.3
Systolic BP (mmHg)	EG CG	106.84 ± 10.15 110.80 ± 12.80	103.64 ± 8.54 ^*,*#*^ 111.45 ± 11.20	−3.0 ^†^ +0.6
Diastolic BP (mmHg)	EG CG	74.29 ± 8.43 76.46 ± 10.50	70.84 ± 7.30 ^*,*#*^ 78.27 ± 9.93	−4.6 ^†^ +2.3
FVC (L)	EG CG	3.32 ± 0.52 3.11 ± 0.43	3.44 ± 0.51 ^*,*#*^ 3.10 ± 0.41	+3.6 ^†^ −0.3
FEV_1_ (L)	EG CG	2.63 ± 0.43 2.59 ± 0.34	2.74 ± 0.42 ^*,*#*^ 2.60 ± 0.32	+4.2 ^†^ +0.4

where * *p* < 0.05 statistically significant difference before and after the intervention program in the EG, # *p* < 0.05 statistically significant difference between the EG and the CG in the post-training measurement, and ^†^ *p* < 0.01 statistically significant difference between the EG and the CG in the percent change. BP, blood pressure; CG, control group; EG: exercise group; FVC, forced vital capacity; FEV_1_, forced expiratory volume in 1 s.

**Table 5:** Flexibility and balance values in the exercise and control group pre and post training (mean ± SD).

Variables	Group	Pre Training	Post Training	Mean % Change
Flexibility—Sit-and-reach test (cm)	EG GG	24.61 ± 6.43 24.67 ± 6.35	27.86 ± 6.36 ^*,#^ 24.00 ± 6.38	+12.2 ^†^ −2.7
Static balance—Right leg (s)	EG CG	56.92 ± 40.28 56.91 ± 40.16	108.82 ± 55.64 ^*,#^ 56.18 ± 39.93	+51.1 ^†^ −1.3
Static balance—Left leg (s)	EG CG	48.73 ± 39.80 47.77 ± 39.62	101.73 ± 59.34 ^*,#^ 48.78 ± 39.30	+50.4 ^†^ +2.1
Dynamic balance—TUG test (s)	EG CG	4.90 ± 0.47 4.90 ± 0.46	4.44 ± 0.37 ^*,#^ 4.91 ± 0.47	−10.3 ^†^ +0.2

where * *p* < 0.001 statistically significant difference before and after the intervention program in the EG, # *p* < 0.01 statistically significant difference between the EG and the CG in the post-training measurement, and ^†^ *p* < 0.001 statistically significant difference between the EG and the CG in the percent change. CG: control group; EG: exercise group; TUG test: timed up-and-go test.

**Table 6:** Strength values in the exercise and control groups pre and post training (mean ± SD).

Variables	Group	Pre Training	Post Training	Mean % Change
Maximal HG preferred hand (kg)	EG CG	31.11 ± 5.48 31.28 ± 5.54	35.36 ± 5.35 ^*,#^ 30.58 ± 5.27	+12.2 ^†^ −2.3
Maximal HG non-preferred hand (kg)	EG CG	30.17 ± 5.09 30.22 ± 5.05	33.61 ± 5.81 ^*,#^ 29.61 ± 4.97	+10.0 ^†^ −2.1
Sit-ups (reps)	EG CG	14.50 ± 9.78 14.67 ± 9.82	32.56 ± 13.86 ^*,#^ 14.22 ± 9.08	+55.9 ^†^ −2.4
Push-ups (reps)	EG CG	12.17 ± 7.16 12.28 ± 6.94	21.00 ± 7.81 ^*,#^ 12.72 ± 6.75	+43.9 ^†^ +1.5

where * *p* < 0.001 statistically significant difference before and after the intervention program in the EG, # *p* < 0.01 statistically significant difference between the EG and the CG in the post-training measurement, and ^†^ *p* < 0.001 statistically significant difference between the EG and the CG in the percent change. CG, control group; EG: exercise group; HG: handgrip strength.

**Table 7:** Cardiorespiratory fitness in the exercise and control groups pre and post training (mean ± SD).

Variables	Group	Pre Training	Post Training	Mean % Change
HR rest (beats/min)	EG CG	83.50 ± 8.86 83.33 ± 8.81	73.72 ± 8.50 ^*,#^ 84.44 ± 8.79	−13.8 ^†^ +1.3
HR test (beats/min) Stage 1°	EG CG	114.78 ± 6.12 114.33 ± 6.09	105.06 ± 8.45 ^*,#^ 115.72 ± 6.72	−9.8 ^†^ +1.2
Stage 2°	EG CG	132.11 ± 9.04 131.89 ± 8.71	122.67 ± 11.57 ^*,#^ 132.83 ± 9.44	−8.2 ^†^ +0.7
Stage 3°	EG CG	145.06 ± 7.46 145.88 ± 7.47	137.82 ± 11.40 ^*,#^ 144.50 ± 7.55	−6.4 ^†^ −0.9
HR rec (beats/min) 1st min	EG CG	113.67 ± 11.28 113.56 ± 11.11	100.83 ± 11.44 ^*,#^ 114.06 ± 11.30	−13.3 ^†^ +0.4
RPE (Borg scale) Stage 1°	EG CG	9.22 ± 2.05 9.50 ± 2.19	7.44 ± 1.62 ^*,#^ 9.11 ± 2.20	−24.7 ^†^ −1.5
Stage 2°	EG CG	11.83 ± 3.05 11.72 ± 2.97	9.50 ± 2.64 ^*,#^ 12.17 ± 2.94	−25.9 ^†^ +2.6
Stage 3°	EG CG	13.50 ± 3.12 13.19 ± 3.15	11.12 ± 2.96 ^*,#^ 13.75 ± 3.44	−25.5 ^†^ +2.4
VO_2max_ estimation (mL/kg/min)	EG CG	36.50 ± 3.1 36.89 ± 2.5	41.2 ± 3.0 ^*,#^ 36.5 ± 2.4	+11.4 ^†^ −1.1

where * *p* < 0.001 statistically significant difference before and after the intervention program in the EG, # *p* < 0.01 statistically significant difference between the EG and the CG in the post-training measurement, and ^†^ *p* < 0.001 statistically significant difference between the EG and the CG in the percent change. HRrest: heart values at rest in a sitting position, HR test: heart values during the submaximal exercise, HR rec: heart values following the submaximal exercise, and RPE test: the rating of perceived exertion during the submaximal exercise. VO_2max_ was estimated using the following equation: VO_2max_ = 15.1 + 21.8 (speed in mph) − 0.327 (heart rate in bpm) − 0.263 (speed × age in years) + 0.00504 (heart rate in bpm × age in years) + 5.98 (gender: female = 0, male = 1).

## Data Availability

Data are unavailable due to privacy or ethical restrictions.
